# Substrate-based inhibitors exhibiting excellent protective and therapeutic effects against Botulinum Neurotoxin A intoxication

**DOI:** 10.1038/srep16981

**Published:** 2015-11-20

**Authors:** Jiubiao Guo, Jinglin Wang, Shan Gao, Bin Ji, Edward Waichi Chan, Sheng Chen

**Affiliations:** 1 Shenzhen Key lab for Food Biological Safety Control, Food Safety and Technology Research Center, Hong Kong PolyU Shen Zhen Research Institute, Shenzhen, P. R. China; 2 State Key Lab of Chirosciences, Department of Applied Biology and Chemical Technology, The Hong Kong Polytechnic University, Hung Hom, Kowloon, Hong Kong; 3 State Key Laboratory of Pathogen and Biosecurity, Beijing Institute of Microbiology and Epidemiology, Fengtai District, Beijing, People’s Republic of China

## Abstract

Potent inhibitors to reverse Botulinum neurotoxins (BoNTs) activity in neuronal cells are currently not available. A better understanding of the substrate recognition mechanism of BoNTs enabled us to design a novel class of peptide inhibitors which were derivatives of the BoNT/A substrate, SNAP25. Through a combination of *in vitro*, cellular based, and *in vivo* mouse assays, several potent inhibitors of approximately one nanomolar inhibitory strength both *in vitro* and *in vivo* have been identified. These compounds represent the first set of inhibitors that exhibited full protection against BoNT/A intoxication in mice model with undetectable toxicity. Our findings validated the hypothesis that a peptide inhibitor targeting the two BoNT structural regions which were responsible for substrate recognition and cleavage respectively could exhibit excellent inhibitory effect, thereby providing insight on future development of more potent inhibitors against BoNTs.

Botulinum neurotoxins (BoNTs) are the causative agents of botulism which specifically interfere with synaptic vesicle fusion and neurotransmitter release in nerve cells^1^,^2^. Synthesized as a 150 kDa single chain protein, BoNT is subsequently cleaved into a di-chain linked by a disulfide bond between its 50 kDa light chain (LC) and 100 kDa heavy chains (HC), which may be further segregated into three functional domains: an N-terminal catalytic domain (light chain, LC), an internal translocation domain (heavy chain, HCT), and a C-terminal receptor binding domain (heavy chain, HCR)^3^. BoNTs are known to inhibit exocytosis by specifically cleaving one of the three SNARE (soluble N-ethylmaleimide sensitive factor attachment protein receptors) proteins: SNAP25 (soluble NSF attachment protein of 25k Da), VAMP2 (vesicle associated membrane protein 2) and syntaxin 1a. Formation of the protein complex by these three proteins, known as the SNARE complex, is the driven force of mammalian neuronal exocytosis^4^. To date, seven serotypes of BoNTs (designated as BoNT/A-G) that cleave specific residues on one of the three SNARE proteins have been identified^2^,^5^,^6^,^7^.

It is well known that muscles will regain function upon clearance of the BoNTs infected neuronal cells. The reversible nature of BoNTs intoxication has enabled these compounds to be transformed from deadly agents to novel therapeutic drugs for treatment of a range of neuromuscular conditions^8^,^9^,^10^,^11^,^12^,^13^,^14^,^15^,^16^,^17^. However, with the persistent problems of intoxication risk, mal-functional use and drug overdose in extensive clinical applications, efforts to develop safer and more effective BoNTs-based therapeutic approaches have intensified. Vaccines and monoclonal antibodies^18^,^19^,^20^, including *E.coli*–based recombinant subunit vaccine^21^,^22^,^23^ and human-derived polyclonal- and neutralizing monoclonal- antibodies that block the entry of BoNTs into nerve cells, have been developed or intensively studied. The major limitation regarding the use of vaccine and antibodies is that their effectiveness will be drastically reduced after the entry of BoNTs into nerve cells. Therefore, potent inhibitors that that inactivate BoNTs activity within the nerve cells are urgently needed to provide therapeutic effects under such situations.

Efforts have previously been made to develop small molecule inhibitors targeting the active site of BoNTs using various approaches, including direct compound library screening, computer-aided small molecular design, and screening of natural compounds. However, the most potent small molecule inhibitors that have been identified so far only exhibited inhibitory effects at μM range. In addition, their affinity (*K*_*m*_) towards BoNTs is similar to that of BoNT substrates; hence they have no advantage in being used as a potent inhibitor^24^,^25^,^26^,^27^,^28^,^29^. Attempts to develop natural product-based small inhibitors that target the binding domain of BoNTs also failed^29^. Technical difficulties associated with development of small molecule inhibitors for BoNTs are probably due to the unique substrate recognition mechanisms of such compounds, in which an extended recognition and cleavage region in the substrate molecule is required for efficient hydrolysis mediated by BoNTs^30^,^31^,^32^,^33^,^34^. Nevertheless, small peptide inhibitors targeting the active site of BoNTs, developed through structure and substrate-based design, have been studied, with the most promising peptide inhibitors exhibiting a Ki as low as nM range. The inhibition mechanism of these peptide inhibitors is through competing with the corresponding substrate in interaction with the active site of BoNTs^35^,^36^,^37^,^38^,^39^. These studies, therefore, offer a promising hint for development of effective peptide-based BoNTs inhibitors. However, the fact that the peptide inhibitors produced in these previous studies only target the active site of BoNTs has prevented development of compounds with higher potency.

Several previous studies indicated that interactions between LC/A (light chain of BoNT/A)-SNAP25 were not optimal, and that mutation at specific sites could improve both substrate binding and catalysis^30^,^40^. These findings provide valuable information which supports the hypothesis that peptide inhibitor targeting both active site and binding regions of BoNTs could dramatically increase its affinity and potency. In the present study, we reported the development of potent peptide inhibitors of BoNT/A which exhibited nM inhibition effect both *in vitro* and *in vivo*. Most importantly, these novel substrate-based inhibitors could provide full protection against 4XLD_50_ challenge by BoNT/A without detectable toxicity, representing the most effective BoNT inhibitors produced to date. Our findings infer that BoNT substrate-based inhibitors exhibit huge potential in future development of effective therapeutic agents against BoNT/A intoxication.

## Results

The regions distal to cleave site in SNAP25 contribute significantly to the substrate affinity toward LC/A in the multi-step substrate recognition process^33^,^40^,^41^,^42^. In addition, our previous study showed that LC/A could bind to another region of SNAP25, namely SNAP25(80–110), facilitating the recognition and cleavage of SANP25 on neuronal cell membrane^42^. Based on these findings and our data on saturation mutagenesis mapping of SNAP25, we developed potent substrate based inhibitors for BoNT/A since small molecule inhibitors lacking the distal binding site did not show a promising level of potency. In our design, we used SNAP25(80–196), which included both binding sites of LC/A, as backbone for the development of various forms of inhibitors.

### Screening for SNAP25 sites that contribute to higher binding to LC/A

Previous findings suggested that the LC/A-SNAP25 interactions could be optimized^30^,^40^. In this work, by analyzing the co-crystal structure of LC/A-SNAP25 (PDB ID: 1XTG), and on the basis of our previous understanding of the mechanism of substrate recognition of LC/A, we shortlisted some residues in SNAP25 that could enhance its affinity to LC/A^33^,^41^. These residues, including H^162^, R^180^, E^183^, D^186^, T^190^, E^194^ and M^202^, could potentially interact with LC/A in a format of SNAP25/LCA: H^162^/K^340^, R^180^/Y^144^, E^183^/P^25^, D^186^/O atom of P^25^ and N^26^, T^190^/F^168^, E^194^/P^239^ and M^202^/L^200^, Y^250^/F^369^. These interactions may not be optimal and may affect the binding of SNAP25 to LC/A. We therefore created different substitutions in these residues with the hope of achieving optimal interactions at these sites. In order to screen the effect of SNAP25 substitution on LC/A binding affinity, we used a LC/A activity assay to screen for the mutations that resulted in higher binding affinity to LC/A. The idea is that, with the LC/A-SNAP25 active site recognition efficiency unchanged, an increase in binding affinity of SNAP25 to LC/A could subsequently result in an increase in the efficiency of LC/A cleavage of SNAP25. By the use of this assay, we observed that amino acid changes at various sites of SNAP25 could enhance the cleavage efficiency of LC/A by 10 ~ 20 folds, with SNAP25 (H^162^D) and (R^180^L) being the most efficient substitutions (**ST 1**). The improved binding affinity between LC/A and SNAP25(H^162^D) could be due to the optimal interaction between K^340^ of LC/A and D^162^ of the SNAP25 mutant (SF 1A). In Wt-SNAP25, H^162^ of SNAP25 may repel K^340^ of LC/A, eliciting a negative impact at this interaction site (SF 1B). For R^180^ of SNAP25, the possible interacting residues in LC/A are S^143^ and Y^144^ (SF 1C), which form a hydrophobic pocket. The finding that the substitution R^180^L could help optimize the interaction between SNAP25 and LC/A (SF 1D) was consistent with our data that SNAP25 (R^180^L) increased LC/A substrate cleavage efficiency. Double mutants of SNAP25, such as SNAP25 (H^162^D, R^180^L) and SNAP25 (T^190^V, M^202^F), however, did not exhibit enhanced cleavage efficiency by LC/A, suggesting that optimizing interaction at multiple sites simultaneously did not produce a synergistic effect (**ST 1**).

### Development of inhibitors using SNAP25 based peptides with enhanced affinity

The tetra-peptide RRGF was reported to exhibit an IC_50_ of 0.9 uM and a K_i_ as low as 358 nM^43^ when using SNAPtide as substrate (a 17-residue synthetic peptide corresponding to the residues of SNAP25 (187–203)). However, when using SNAP25(141–206) as substrate, the IC_50_ was about 1000-fold higher, suggesting that RRGF could effectively inhibit the binding of SNAPtide to the active site of LC/A, whereas its inhibition to SNAP25(141–206) was not effective due to the absence of a LC/A binding site in SNAP25. However, this tetra-peptide is still the best peptide to constitute the C-terminus part of our peptide inhibitor. Therefore, SNAP25(80–197)-RRGF and SNAP25(80–197)- WTKFL were used as the backbone of the inhibitor and designated as R1 and R2 respectively (Table 1). In addition, our previous study also showed that substituting the P1’ site of VAMP2 by Cys residue could covert BoNT substrate into a weak inhibitor. Therefore, SNAP25 (80–196)-C was also used as another peptide inhibitor backbone and designated as R197C (Table 1). The IC_50_ of these three inhibitors, R1, R2 and R197C, were determined to be 17.15 μM, 15.71 μM and 2.22 μM respectively and the Ki were 13.52 μM, 12.39 μM and 1.75 μM respectively (Table 2). We then incorporated different amino acid substitutions that could enhance the binding of SNAP25 peptides into these three inhibitors, including H^162^D, R^180^L, E^183^L, D^186^H, T^190^V, H^162^D/T^190^V. Inhibitor R1 (R^180^L) displayed the highest inhibitory effect, with a IC_50_ of 0.28 μM and K_i_ of 0.22 μM (Table 2). Inhibitor, R1 (H^162^D) and R1 (H^162^D, T^190^V) had a slightly higher IC_50_ and K_i_ than R1(R^180^L) (**ST 2**). Inhibitor R2 (H^162^D) exhibited IC_50_ of 1.11 μM and K_i_ of 0.88 μM (**ST 2**) and R2 (T^190^V) exhibited almost the same inhibitory effect (Table 2). However, inhibitor R2 (R^180^L), did not enhance the inhibitory effect. For the R197C type of inhibitor, R197C (D^186^H) exhibited IC_50_ of 0.28 μM and K_i_ of 0.22 μM (**ST 2**).

Further analysis of the modeled complex structures of LC/A-R1 and LC/A-R2 showed that replacing RRGF at the C-terminus of the inhibitor by RGF could fit these inhibitors better in LC/A (SF 2), particularly in the active site. Additional inhibitors, such as SNAP25(80–196)-RGF, which was designated as R1-RGF, plus other derivatives, were generated (Table 1). Inhibitor R1-RGF exhibited a very low IC_50_ of 0.0021 μM and a Ki of 0.0017 μM (Table 2). Inhibitor R1-RLF exhibited an IC_50_ of 0.93 μM and a Ki of 0.83 μM (**ST 2**). The more potent inhibitory effects exhibited by these inhibitors, when compared to the one with RRGF as C-terminus, may be due to a better fit of the C-terminus of inhibitor into the active site of LC/A, as evidenced by examination of the structure of LC/A-RRGF (PDB ID: 3QW5).

### Inhibition of LC/A by SNAP25 based inhibitors in cell model

**Figure 1:**
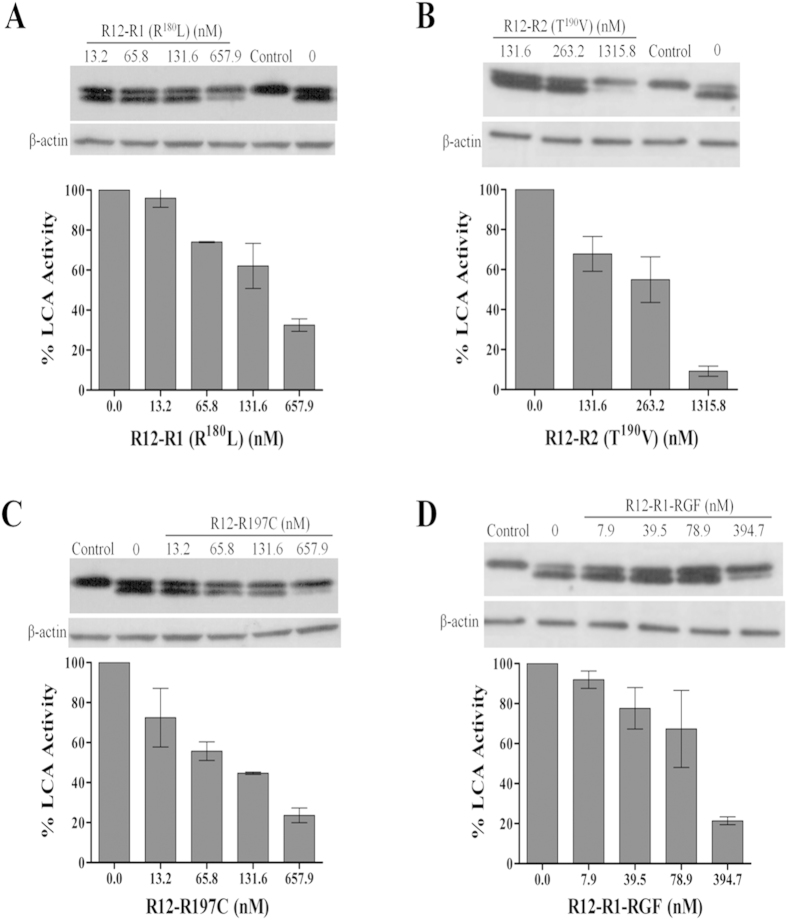
Inhibition of LC/A activity by different peptide inhibitors in Neuro-2a cells. 0.5 μg pEGFP-C3-LC/A (1-425) plasmid were transfected into Neuro-2a cells; 4 h post-transfection, the cells were incubated with indicated concentrations of R12-coupled peptide inhibitors, (**A**) R12-R1 (R^180^L), (**B**) R12-R2 (T^190^V), (**C**) R12-R197C and (**D**) R12-R1-RGF, in complete DMEM medium. The inhibition of SNAP25 cleavage by LC/A was scored by Western-blotting. The amount of LC/A in each treatment was normalized by quantifying the total green fluorescent signals, which were found to be at the same level in all treatment groups, suggesting that a similar amount of LC/A was expressed in each treatment (data not shown).

In order to further investigate the *in vivo* inhibitory effects of the peptide inhibitors developed as described above, we coupled oligoarginines (R12) with the four most promising peptide inhibitors, namely R1 (R^180^L), R2 (T^190^V), R197C and R1-RGF, and performed cell-based inhibition assays. Consistent with our *in vitro* results, these four peptide inhibitors exhibited high LC/A inhibitory effect *in vivo* as well. About 0.7  μM R12-R1 (R^180^L) and R12-R197C were found to inhibit >60% activity of LC/A, with the latter exhibiting slightly higher inhibitory effects on LC/A *in vivo* (Fig. 1A,C); however, the *in vitro* inhibition effect of R1 (R^180^L) was about 10-fold higher than that of R197C (Table 2). More than 80% LC/A activity was inhibited by about 1.3 μM R12-R2 (T^190^V) (Fig. 1B); for R12-R1-RGF, the concentration required to inhibit >80% LC/A activity was as low as 0.4 μM (Fig. 1D). In all experiments, the amount of LC/A in each treatment was normalized by quantifying the total green fluorescent signals, which were found to be very similar in all treatment groups, suggesting that a similar amount of LC/A was expressed in each treatment (data not shown).

### Full protection of BoNT/A intoxication by SNAP25 inhibitors in mice

**Figure 2:**
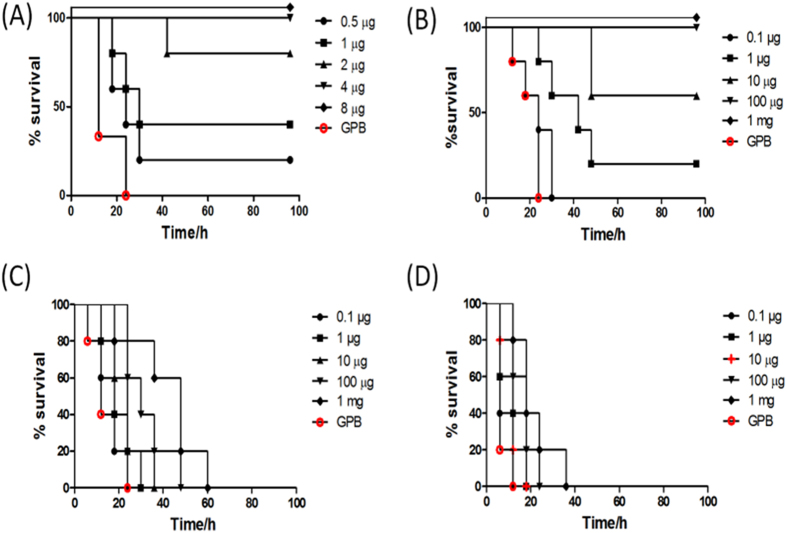
Protection of mice against different dosages of BoNT/A challenge by SNAP25-based inhibitor. BoNT/A at dosage of 2 × LD_50_ (**A**), 4 × LD_50_ (**B**), 8 × LD_50_ (**C**) and 16 × LD_50_ (**D**), were mixed with different amounts of peptide-based inhibitors, R12-R1-RGF, 30min prior to inoculation into mice. The death rate of the test animals was recorded overtime. GPB refers to control group.

To further examine the inhibitory effect of SNAP25 based inhibitors to BoNT/A intoxication *in vivo*, we performed protection assays using inhibitor R12-R1-RGF. Our data showed that a dose of 2 μg R12-R1-RGF or higher could fully protect the challenge of 2 × LD_50_ of BoNT/A (Fig. 2A), and that a dose of 10 μg or higher could protect 4 × LD_50_ challenge by BoNT/A (Fig. 2B). At higher doses (8 × LD_50_ and 16 × LD_50_) of BoNT/A challenge, the protective effects of R12-R1-RGF were not apparent, but the application of inhibitors at 10 μg or higher could delay the killing of mice by BoNT/A (Fig. 2C,D). Most importantly, application of up to10mg of R12-R1-RGF did not cause any observable toxicity in mice.

## Discussion

The dual status of Botulinum Neurotoxin as causative agent of human botulism and bioterrorism weapon, as well as the most widely used protein therapeutic agent for neuromuscular disorders, has greatly elicited a need for development of small molecule inhibitors in the past decade. However, due to the unique substrate recognition mechanism of these classic toxins, small molecule inhibitors that specifically bind to the active site of BoNT are not readily designed and synthesized. The interaction between the toxin and its substrate at the distal site renders active site inhibition ineffective. This is especially evidenced from our data which showed that the tetra-peptide RRGF and its derivatives exhibited very potent inhibitory effects on LC/A cleavage of SNAPtide, whereas their ability to inhibit LC/A cleavage of SNAP25 (141–206) decreased by 1000 folds due to the presence of an additional LC/A binding site in the region of SNAP25 (141–180). Therefore, development of peptide inhibitors that constitute both the binding and substrate recognition site of SNAP25 may offer the most effective inhibition^41^.

Another unique feature of BoNT/A substrate interaction is that it can recognize an additional binding site at the region of SNAP25 (80–110), thereby facilitating LC/A to bind to SNAP25 in neuronal cells. This additional binding may offer LC/A an advantage to compete with syntaxin to initiate its substrate recognition process since in syntaxin-SNAP25 or SNARE complex, the LC/A substrate binding region SNAP25(141–206) is occupied to form the SNARE complex. Therefore the free region SNAP25(80–110) in the complex form of SANP25 becomes the first target when LC/A comes into contact with SNAP25, which is then followed by further substrate recognition and cleavage. This binding site could also contribute to high affinity binding of LC/A to SNAP25. Therefore, region of SNAP25(80–196) was selected as the backbone of the inhibitor.

In addition to the binding site, the active site architecture of the inhibitor is also important as incorporation of known active site inhibitor RRGF and its derivatives dramatically increased the inhibitory effect. In this work, structural analysis of the effects of inhibitors R1 and R2 to LC/A showed that the C-terminal RRGF may not fit the active site well. Therefore we replaced the C-terminal part with RGF and its derivatives (Fig. 2), and found that R1-RLF exhibited an increased potency, with an IC_50_ of 0.93 μM, and R1-RGF exhibited an even more dramatically improved potency, with an IC_50_ of 0.0021 μM. These compounds are the most potent inhibitors that have been reported in the literature to date. Most importantly, R1-RGF not only exhibited potent inhibition of LC/A activity in cell model, but also displayed full protection against 4 × LD_50_ LC/A challenge in mice, suggesting a huge potential of this inhibitor in being applied for BoNT/A intoxication treatment. It should also be stressed that this inhibitor is the first to display full protection against LC/A challenge.

In conclusion, we have developed a series of SNAP25 based inhibitors with nanomolar inhibitory strength both *in vitro* and *in vivo*. Most importantly, these inhibitors for the first time showed full protection of mice intoxicated with 4XLD_50_ of BoNT/A. This is, by far, the first BoNT/A inhibitor with excellent clinical application potential.

## Materials and Methods

### Plasmid construction and protein purification

LC/A and SNAP25 were constructed and purified as described previously. Briefly, the DNA fragment encoding LC/A (1-425) was sub-cloned into pET-15b vector, transformed into *Escherichia coli* Bl21 (DE3) RIL (Stratagene). SNAP25 (141–206) was sub-cloned into pGEX-2T vector for glutathione S-transferase (GST) tagged fusion protein, which was used as substrate in subsequent functional assays. In addition, DNA fragments that encode the SNAP25 based peptide inhibitors were sub-cloned into pET-15b vector for His tagged fusion protein, and then transformed into *Escherichia coli* Bl21 (DE3) for expression. All proteins were purified as previously described^30^.

### Development of high affinity peptides for LC/A

In order to produce highly potent LC/A inhibitors, site-directed mutagenesis was performed, using SNAP25 (141–206) as backbone, to mutagenize sites which were selected based on the co-crystal structure of LC/A-SNAP25 (PDB ID: 1XTG) and previous understanding of LC/A and SNAP25 recognition mechanism^33^,^41^. The newly created mutations were confirmed by sequencing. All proteins were purified and subjected to activity analysis as previously described^30^.

### Development of potential LC/A inhibitors with high inhibition efficiency

Kumar *et al.* reported a potential LC/A tetra-peptide inhibitor, RRGF, with an IC_50_ of 0.9 uM and a K_i_ as low as 358 nM, but the substrate used in this previous study was a 17-residue synthetic peptide corresponding to residues of SNAP25 (187–203)^43^. In addition, we have previously found that LC/A almost could not cleave the SNAP25 Q^197^C mutant (unpublished data). Based on these findings, we designed the following peptides: 1) R197C, the backbone of which is SNAP25 (80–196), with a C added at the C-terminal; 2) R1, the backbone of which is SNAP25 (80–196), plus CRRGF at the C terminal; 3) R2, the backbone of which is also SNAP25 (80–196), plus CWTKFL at the C terminal; and 4) R1-RXF, the backbone of which is R1, but with the first R deleted from the RRGF tetra-peptide at the C-terminal, and the G substituted by some other amino acids. Secondly, the sites screened above with enhanced LC/A affinity were incorporated into the first three backbones of the peptides to further analyze their *in vitro* inhibitory effects on LC/A by linear velocity assays. Briefly, the reactions were performed in 10 μl volume. Appropriate concentration of recombinant LC/A was first incubated with different concentrations of peptide inhibitors on ice for 20 minutes in 10 mM Tris-HCl (pH 7.6) and 20 mM NaCl. Indicated concentration of SNAP25 was then added into the reaction mixture, which was incubated for another 20 min at 37 °C, stopped by adding sample loading buffer, and heated for 5 min. Samples were subjected to SDS-PAGE analysis. The IC_50_ of peptide inhibitors were calculated by densitometry and the corresponding K_i_ was calculated by the formula of IC50 = (1+[S]/K_M_).

### Cell-based inhibition of potential peptide-based inhibitors

In order to deliver peptide-based inhibitors into target cells, oligoarginines (R12) coupled with multiple Serine-Alanine linker sequence were directly added to the N-terminus of the most potential peptide inhibitors through recombinant DNA technology. Neuro-2a mouse neuroblastoma cells (ATCC) were maintained in DMEM medium with 10% newborn calf serum and 1% P/S antibiotics to prevent contamination, then grown on cell-culture dishes at 37 °C under 5% CO_2_ until approximately 80% confluence. 0.5 μg pEGFP-C3-LC/A (1-425) plasmid were firstly transfected into Neuro-2a cells using Lipofectamine LTX plus (GIBCO/BRL) as suggested by manufacturer; 4 h post-transfection, the cells were incubated with indicated concentrations of R12-coupled peptide inhibitors in complete DMEM medium (with 10% FBS and 1% P/S added). After 48 h incubation, cells were harvested and washed by PBS, lysed by 1 × RIPA, with 1 mM PMSF added, then left on ice for 10 min. Cells were scraped and spun down at 14,000 rpm for 10 min at 4 °C, the total protein in the supernatant was quantified by the Bradford method (Bio-Rad). Samples were subjected to SDS-PAGE and western blotting, the cleavage of SNAP25 was determined by probing with anti-SNAP25 antibody (SMI 81, abcam) and the amount of SNAP25 cleavage was determined by densitometry. The IC_50_ and K_i_ were measured as described above.

### Evaluation of selected peptide inhibitors in mice model

In order to further test the inhibitory effect of the most promising peptide inhibitors, a mouse model test was performed as previously reported^44^. Six-week-old female BALB/c mice were used in all the assays. For LD_50_ determination, raw BoNT/A holotoxin was serially diluted in GPB buffer (0.05 M sodium phosphate, pH 6.8, 0.2% gelatin). The mice were divided into six groups with eight mice per group. The five experimental groups of mice were injected intraperitoneally (i.p.) through lateral tail vein with 0.2 ml of a solution of the toxin (0.875–14 ng). The control group was treated only with 0.2 ml GPB buffer. Animals were observed for signs of botulism and death continuously for a period of 96 h, with record being taken at each 6 h interval. The LD_50_ was determined based on the Karber’s method. The toxicity test of the selected peptide inhibitor was measured as described above, except that mice were randomly divided into four groups with four mice per group. The survival rate and death was recorded every 6 h during a time period of 96 h. For the *in vivo* inhibition tests, the most promising peptide inhibitor was selected based on the aforementioned cell-based assays. The mice were randomly divided into six groups with eight mice per group. Before i.p. injection, desired concentrations of peptide inhibitor were mixed with different amounts of the prepared BoNT/A samples (ranged from 1 × LD_50_ to 16 × LD_50_), incubated at 37 °C for 30 min, 0.2 ml of which was injected into mice. The control group was treated with 0.2 ml GPB buffer. All mice were examined for several days and the survival, behavior, breath rates and signs of expression of botulism symptoms were recorded at 6 h interval.

### Ethics Statement

All experimental mice were obtained from the Laboratory Animal Center, Academy of Military Medical and Sciences with free access to food and water. All experiments were conducted strictly in accordance with the guidelines of the Chinese Association for the Accreditation of Laboratory Animals Care (CAALAC), including the relevant local animal welfare bodies in China. The permit number of the animal work was SCXK-(JUN) 2013–005; the work was approved by the animal ethics committee of Beijing Institute of Microbiology and Epidemiology.

## Additional Information

**How to cite this article**: Guo, J. *et al.* Substrate-based inhibitors exhibiting excellent protective and therapeutic effects against Botulinum Neurotoxin A intoxication. *Sci. Rep.* **5**, 16981; doi: 10.1038/srep16981 (2015).

## Supplementary Material

Supplementary Information

## Figures and Tables

**Table 1:** Nomenclature and structure of peptide inhibitors tested in this study.

Peptide inhibitor	Backbone
R197C	SNAP25 (80-196), plus Cys at C-terminal
R1	SNAP25 (80-196), plus CRRGF at C-terminal
R2	SNAP25 (80-196), plus CWTKFL at C-terminal
R1-RXF#	SNAP25 (80-196), plus CRXF at C-terminal

^#^X represents various amino acids used.

**Table 2:** IC_50_ and K_i_ of peptide inhibitors of LC/A (1-425).

Peptide inhibitors	IC_50_¶ (μM)	K_i_ǂ (μM)
RRGF	0.9	0.358Δ
	912.5 ± 0.19	719.57 ± 0.15*
R1	17.15 ± 0.83	13.52 ± 0.17
R1 (R^180^L)	0.28 ± 0.44	0.22 ± 0.35
R2	15.71 ± 0.05	12.39 ± 0.04
R2 (T^190^V)	1.13 ± 0.20	0.89 ± 0.16
R197C	2.22 ± 0.31	1.75 ± 0.24
R1-RGF	0.0021 ± 0.002	0.0017 ± 0.0012

^¶^Average of at least three measurements.

^ǂ^The equation used in the calculation is: K_i_ = IC_50_/(1+[S]/K_M_), and the *K*_*m*_ of LCA (1-425) is 16 uM 30.

^Δ^Data from ref. 43.

^*^Data of the present work.
