# Supplementary material for: Substrate-based inhibitors exhibiting excellent protective and therapeutic effects against Botulinum Neurotoxin A intoxication
Source: Sci Rep. 2015 Nov 20;5:16981. doi: 10.1038/srep16981 (PMC4653808; doi:10.1038/srep16981)
Supplement: Supplementary Information [file srep16981-s1.doc]

**Substrate-based inhibitors exhibiting excellent protective and therapeutic effects against Botulinum Neurotoxin A intoxication**

Jiubiao Guo^1,2^, Jinglin Wang^3^, Shan Gao^3^, Bin Ji^3^, Edward Waichi Chan^1,2^, Sheng Chen^1,2^*

^1^Shenzhen Key lab for Food Biological Safety Control, Food Safety and Technology Research Center, Hong Kong PolyU Shen Zhen Research Institute, Shenzhen, P. R. China

^2^State Key Lab of Chirosciences, Department of Applied Biology and Chemical Technology, The Hong Kong Polytechnic University, Hung Hom, Kowloon, Hong Kong

^3^State Key Laboratory of Pathogen and Biosecurity, Beijing Institute of Microbiology and Epidemiology, Fengtai District, Beijing, People's Republic of China.

*Corresponding author. Tel: (852)-3400-8795; Fax: (852)-2364 9932; Email: [sheng.chen@polyu.edu.hk](mailto:sheng.chen@polyu.edu.hk)

**Keywords:** Botulinum Neurotoxin serotype A, SNAP25, inhibitor, potent, mice model

**Supplementary Table 1 (ST1).** **Cleavage of SNAP25 and its derivatives by LC/A (1-425).**

| SNAP25 and its derivatives | LC/A needed for 90% cleavage of SNAP25/derivatives (ng) | Ratio of LC/A activity  (SNAP25 derivative/wt SNAP25) |
| --- | --- | --- |
| wt SNAP25 (141-206) | 10 | 1 |
| SNAP25 (141-206) (T^190^V) | 1 | 10 |
| SNAP25 (141-206) (T^190^F) | 1 | 10 |
| SNAP25 (141-206) (H^162^D) | 0.5 | 20 |
| SNAP25 (141-206) (R^180^L) | 0.5 | 20 |
| SNAP25 (141-206) (R^180^F) | 1 | 10 |
| SNAP25 (141-206) (M^202^F) | 1 | 10 |
| SNAP25 (141-206) (H^162^D, R^180^L) | 1 | 10 |
| SNAP25 (141-206) (E^183^L) | 1 | 10 |
| SNAP25 (141-206) (E^183^F) | 1 | 10 |
| SNAP25 (141-206) (E^194^L) | 1 | 10 |
| SNAP25 (141-206) (E^194^F) | 1 | 10 |
| SNAP25 (141-206) (D^186^N) | 1 | 10 |
| SNAP25 (141-206) (D^186^H) | 1 | 10 |
| SNAP25 (141-206) (T^190^V, M^202^F) | 1 | 10 |

**Supplementary Table 2 (ST2).** **IC_50_ and K_i_ of LC/A peptide inhibitors.**

| Peptide  inhibitors | IC_50_¶  (μM) | K_i_ǂ  (μM) |
| --- | --- | --- |
| RRGF | 0.9 | 0.358^Δ^ |
|  | 912.5±0.19 | 719.57±0.15* |
| R1 (H^162^D) | 0.64±0.17 | 0.5±0.13 |
| R1 (E^183^L) | 20.40±0.03 | 16.08±0.02 |
| R1 (D^186^H) | 17.69±0.09 | 13.95±0.07 |
| R1 (T^190^V) | 14.72±0.06 | 11.61±0.04 |
| R1 (H^162^D, T^190^V) | 0.60±0.29 | 0.48±0.23 |
| R2 (H^162^D) | 1.11±0.44 | 0.88±0.35 |
| R2 (R^180^L) | 13.96±0.30 | 11.01±0.24 |
| R2 (E^183^L) | 60.86±1.29 | 47.99±1.02 |
| R2 (D^186^H) | 14.47±0.004 | 11.41±0.003 |
| R2 (R^180^L, T^190^V) | 54.40±0.48 | 42.90±0.38 |
| R197C (H^162^D) | 5.24±0.28 | 4.13±0.22 |
| R197C (R^180^F) | 27.09±0.34 | 25.71±0.32 |
| R197C (R^180^L) | 4.78±0.49 | 3.77±0.39 |
| R197C (E^183^L) | 18.75±0.72 | 14.78±0.55 |
| R197C (D^186^H) | 0.28±0.39 | 0.22±0.32 |
| R197C (T^190^V) | 6.36±0.18 | 6.04±0.17 |
| R1-RAF | 20.42±0.09 | 16.10±0.71 |
| R1-RFF | 57.73±1.02 | 45.52±0.80 |
| R1-RIF | 26.88±0.76 | 21.20±0.60 |
| R1-RLF | 0.93±1.02 | 0.73±0.80 |
| R1-RPF | 13.01±2.11 | 10.26±1.67 |
| R1-RWF | 3.72±0.16 | 2.93±0.12 |

¶ Average of at least three measurements.

ǂ The equation used in the calculation is: K_i_=IC_50_ / (1+[S]/K_M_), and the K_M_ of LCA (1-425) is 16uM [^1^](#_ENREF_1).

Δ Data from reference [^2^](#_ENREF_2).

* Data of the present work.


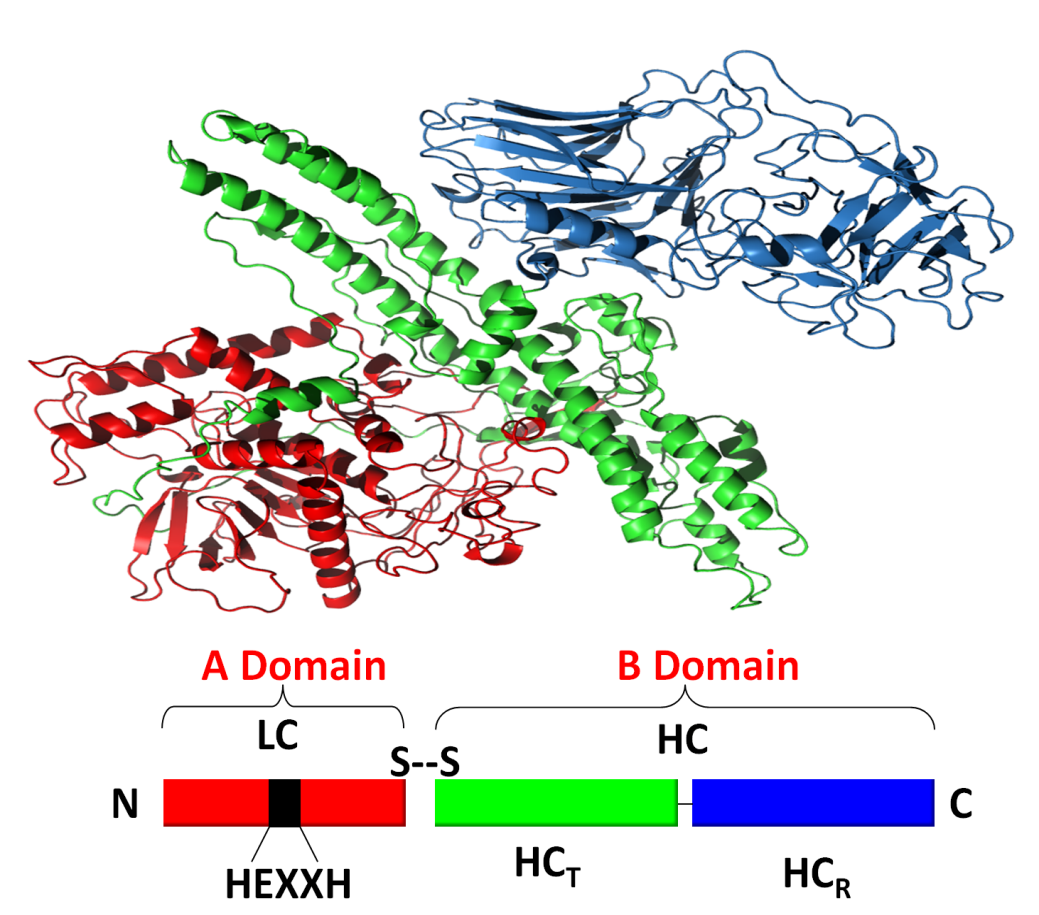


**Supplemental Figure 1 (SF1). Structure and domain organization of Clostridial Neurotoxins.** CNTs are organized into three functional domains: an N-terminal catalytic domain (light chain, LC, red color), an internal translocation domain (heavy chain, HC_T_, green color), and a C-terminal receptor binding domain (heavy chain, HC_R_, blue color).

**
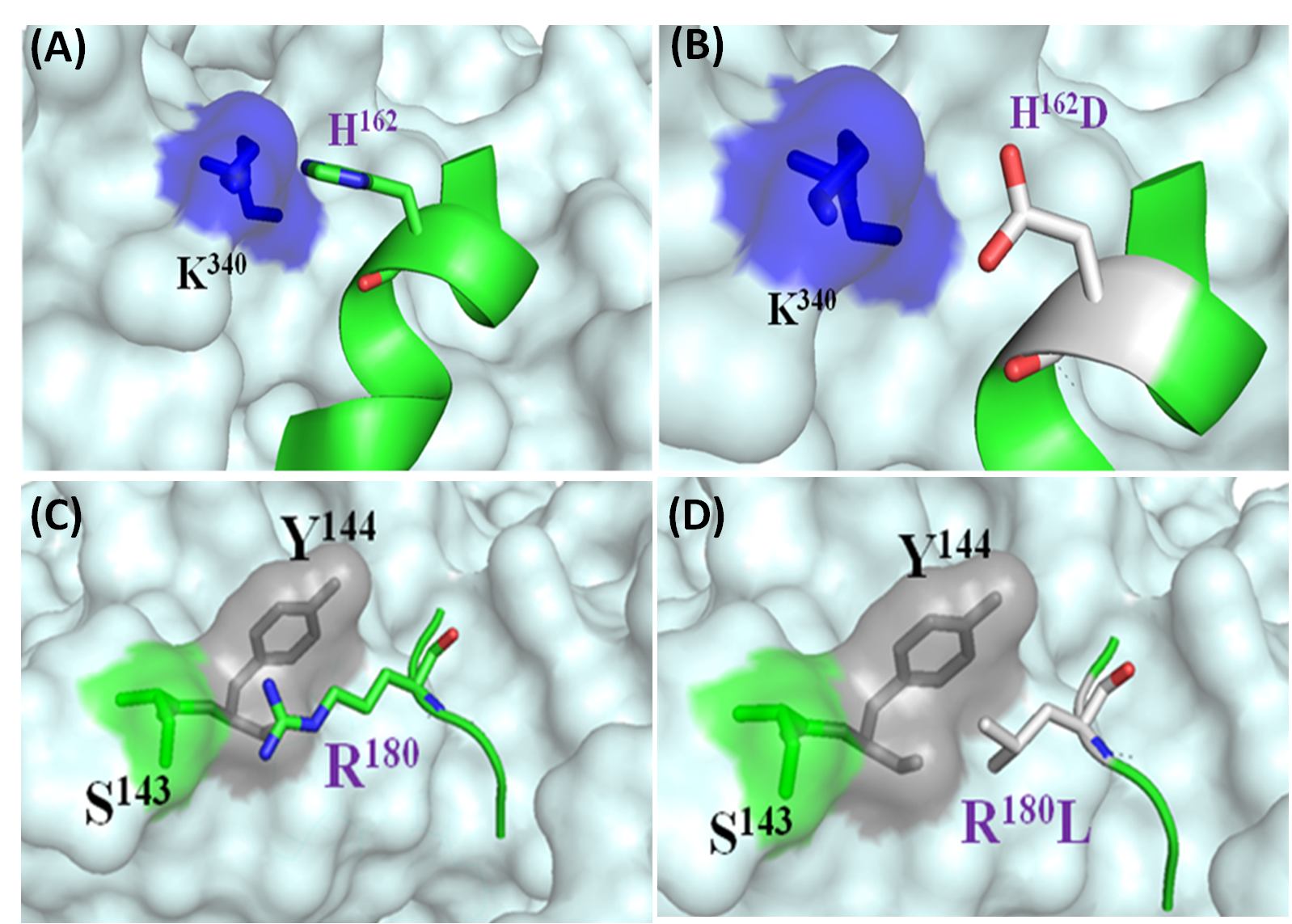
**

**Supplemental Figure 2 (SF2). The two examples illustrate enhanced interactions between SNAP25 and LC/A.** The original possibly interacting residues at the162 site of SNAP25 and the corresponding site in LC/A (A), and the modified residue in SNAP25 which possibly exhibits optimized interactions at the same sites (B). Comparison between the original (C) and modified (D) interactions between the 180 site of SNAP25 and the corresponding sites in LC/A. The LC/A-SNAP25 complex structure was extracted from the PDB bank (ID: 1XTG) with modifications by using the PyMOL software. The LC/A structure was displayed in surface in pale cyan color with specific residues shown in sticks, and SNAP25 shown in cartoon in green, with specific residues also shown in sticks as well. The residues were colored based on the property of the side chain: negatively charged (red), positively charged (blue), hydrophobic (gray) and polar (green).

**
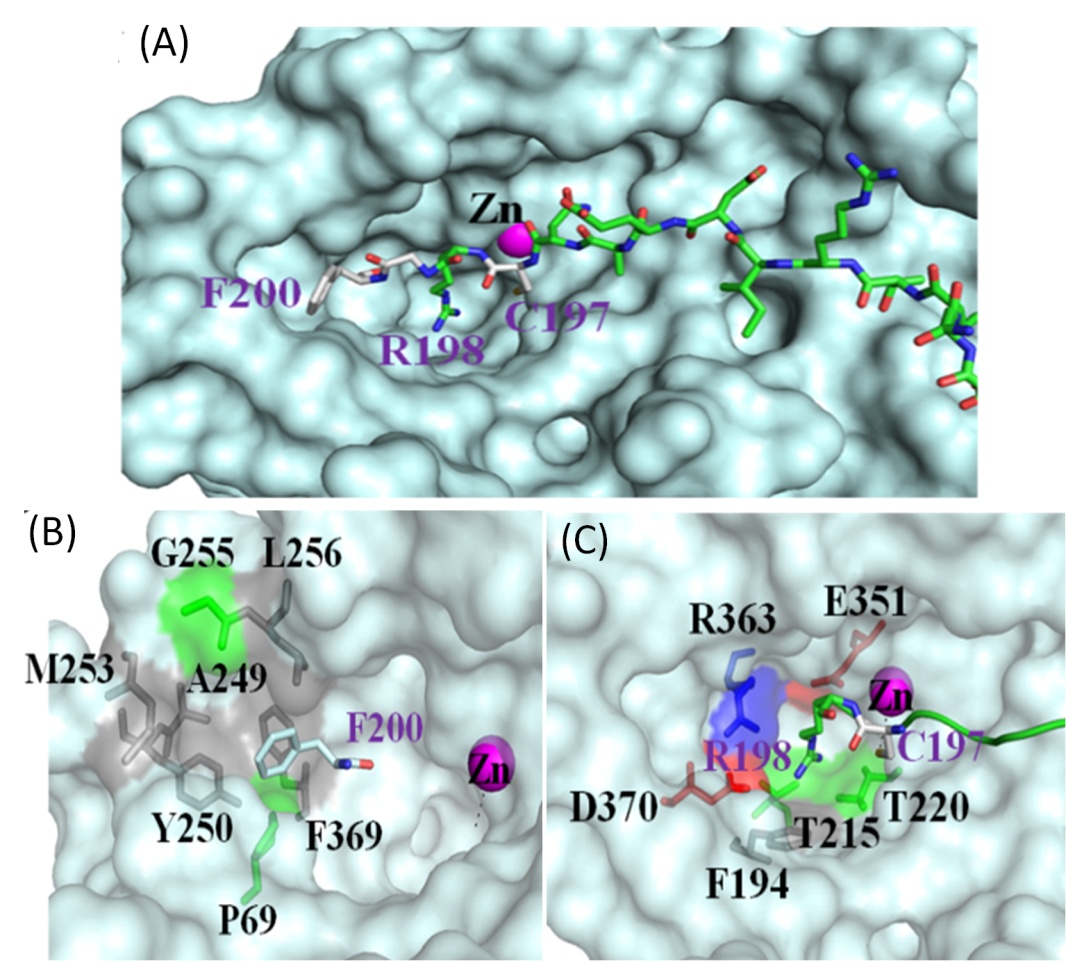
**

**Supplementary Figure 3 (SF3).** **Molecular interactions based on the modeled complex structure of peptide inhibitor and LC/A.** (A) An overall view of the interactions between C197, R198 and F200 of peptide inhibitor with LC/A. (B) The predicted interactions between the F200 from RGF based peptide inhibitors with LC/A. (C) The predicted interactions between the C197 and R198 from RGF based peptide inhibitors with LC/A. The structure of RGF based peptide inhibitors with LC/A was modeled with reference to the crystal structures of LC/A-SNAP25 (PDB ID: 1XTG) and RRGF-LC/A (PDB ID: 3QW5) by using PyMOL software. The LC/A structure was displayed in surface in palecyan color with the specific residues showed in sticks, and the SNAP25 was showed in cartoon in green with the specific residues showed in sticks as well. The residues were colored based on the property of the side chain: negatively charged (red), positively charged (blue), hydrophobic (gray) polar (green). and zinc (magenta sphere).

**Reference:**

1. Chen, S. & Barbieri, J.T. Unique substrate recognition by botulinum neurotoxins serotypes A and E. *The Journal of biological chemistry* **281**, 10906-10911 (2006).

2. Kumar, G., Kumaran, D., Ahmed, S.A. & Swaminathan, S. Peptide inhibitors of botulinum neurotoxin serotype A: design, inhibition, cocrystal structures, structure-activity relationship and pharmacophore modeling. *Acta Crystallographica Section D-Biological Crystallography* **68**, 511-520 (2012).
